# Diagnosis of left ventricular diastolic dysfunction in the setting of acute changes in loading conditions

**DOI:** 10.1186/cc5736

**Published:** 2007-04-11

**Authors:** Philippe Vignon, Vincent Allot, Jérôme Lesage, Jean-François Martaillé, Jean-Claude Aldigier, Bruno François, Hervé Gastinne

**Affiliations:** 1 Medical-surgical Intensive Care Unit, Dupuytren Teaching Hospital, Avenue Martin Luther King, 87000 Limoges, France; 2 Centre de Recherche Clinique, Dupuytren Teaching Hospital, Avenue Martin Luther King, 87000 Limoges, France; 3 University of Limoges, Department of Medicine, Rue du Dr Marcland, 87000 Limoges, France; 4 Department of Nephrology, Dupuytren Teaching Hospital, Avenue Martin Luther King, 87000 Limoges, France

## Abstract

**Introduction:**

Conventional pulsed wave Doppler parameters are known to be preload dependent, whereas newly proposed Doppler indices may be less influenced by variations in loading conditions. The aim of the present study was to evaluate the effects of haemodialysis-induced preload reduction on both conventional and new Doppler parameters for the assessment of left ventricular (LV) diastolic function.

**Methods:**

This prospective observational study was conducted in a medical-surgical intensive care unit (ICU) and nephrology department of a teaching hospital. In total, 37 haemodialysis patients with end-stage renal disease (age [mean ± standard deviation]: 52 ± 13 years) and eight ventilated ICU patients with acute renal failure receiving vasopressor therapy (age 57 ± 16 years; Simplified Acute Physiology Score II 51 ± 17) were studied. Echocardiography was performed before and after haemodialysis. Conventional pulsed wave Doppler indices of LV diastolic function as well as new Doppler indices, including Doppler tissue imaging early diastolic velocities (E' wave) of the septal and lateral portions of the mitral annulus, and propagation velocity of LV inflow at early diastole (Vp) were measured and compared before and after ultrafiltration.

**Results:**

The volume of ultrafiltration was greater in haemodialysis patients than in ICU patients (3.0 ± 1.1 l versus 1.9 ± 0.9 l; *P *= 0.005). All conventional pulsed wave Doppler parameters were altered by haemodialysis. In haemodialysis patients, E' velocity decreased after ultrafiltration when measured at the septal mitral annulus (7.1 ± 2.5 cm/s versus 5.9 ± 1.7 cm/s; *P *= 0.0003), but not at its lateral portion (8.9 ± 3.1 cm/s versus 8.3 ± 2.6 cm/s; *P *= 0.37), whereas no significant variation was observed in ICU patients. Vp decreased uniformly after ultrafiltration, the difference being significant only in haemodialysis patients (45 ± 11 cm/s versus 41 ± 13 cm/s; *P *= 0.04). Although of less magnitude, ultrafiltration-induced variations in Doppler parameters were also observed in haemodialysis patients with altered LV systolic function.

**Conclusion:**

In contrast to other Doppler parameters, Doppler tissue imaging E' maximal velocity measured at the lateral mitral annulus represents an index of LV diastolic function that is relatively insensitive to abrupt and marked preload reduction.

## Introduction

Left ventricular (LV) diastolic properties are a major determinant of LV filling and subsequent stroke volume. On clinical grounds, LV diastolic function is commonly assessed using echocardiography Doppler [1]. Unfortunately, pulsed wave Doppler indices used for the evaluation of LV diastolic properties have long been known to be altered by numerous factors, including loading conditions and heart rate [2-4]. Accordingly, their use to identify LV diastolic dysfunction in clinical settings characterized by abrupt variations in preload or afterload, such as severe sepsis or septic shock [5-7], may be of limited value. Recently, animal and clinical studies have suggested that Doppler tissue imaging (DTI) of the mitral annulus and early diastolic blood flow propagation velocity measured using colour M-mode may constitute parameters of LV diastolic function that are relatively preload independent [8-13].

Haemodialysis provides a unique opportunity to evaluate the effect of a preload reduction on Doppler parameters currently used to assess LV diastolic properties. In this specific setting, recent clinical studies conducted in patients with chronic renal failure have yielded discrepant results [14-19]. The volume of ultrafiltration withdrawn from patients is presumably a major determinant of the sensitivity of Doppler parameters to preload reduction. Accordingly, we sought to evaluate whether the new indices of LV diastolic function provided by DTI of the mitral annulus and colour M-mode were altered by intermittent haemodialysis when performed in two distinct clinical settings: high-volume ultrafiltration in patients with chronic renal failure and lower volume ultrafiltration in ventilated critically ill patients with acute renal failure receiving vasopressor therapy. We also evaluated the potential influence of LV systolic function on variability in Doppler indices induced by haemodialysis.

## Materials and methods

The protocol was approved by the Ethics Committee of the Société de Réanimation de Langue Française, which waived the need of signed informed consent. All patients were informed about the study either at the time of echocardiography (haemodialysis patients) or as early as possible after recovery (ventilated patients).

### Patients

Thirty-seven ambulatory patients with end-stage renal disease (age [mean ± standard deviation] 52 ± 13 years; body mass index 24 ± 5 kg/m^2^) were studied. All patients underwent long-term haemodialysis three times a week for 5.4 ± 5.2 years (range 1 month to 19 years). The aetiology of end-stage renal disease was diabetes in five patients, hypertension in three, lupus erythematosus in three, chronic glomerular disease in 12, chronic interstitial nephropathy in three, or miscellaneous in 11. Eleven patients had ischaemic heart disease and one patient had dilated cardiomyopathy.

Eight ventilated critically ill patients (age 57 ± 16 years; Simplified Acute Physiology Score II 51 ± 17) admitted to the intensive care unit (ICU) with acute renal failure were also enrolled in the study. All patients were sedated so that they could receive volume-controlled ventilation (tidal volume 6.8 ± 1.0 ml/kg; positive end-expiratory pressure 12 ± 3 cmH_2_O; fractional of inspired oxygen adjusted to maintain oxyhemoglobin saturation measured by pulse oxymetry > 92%) and had stable haemodynamics under vasopressor therapy. Patients received a constant dose of vasopressor during the study period (adrenaline [epinephrine; *n *= 6] 0.04 to 0.27 μg/kg per min; noradrenaline [norepinephrine; *n *= 2] 0.04 and 0.19 μg/kg per min). The reason for admission to the ICU was shock of septic origin (*n *= 3) or of other origin (*n *= 4), or a pulmonary oedema (*n *= 1). Only one ICU patient had a history of cardiac disease (ischaemic heart disease).

All patients had normal sinus rythm and no significant (greater than grade I) valvular insufficiency. In all patients, body weight, blood pressure and heart rate were measured before and after haemodialysis. Central venous pressure was also measured before and after the procedure in ICU patients.

### Haemodialysis

In ambulatory patients, haemodialysis was performed over four hours following a standard prescription that was unchanged for at least two months. In ICU patients, the haemodialysis regimen was adapted to both clinical status and metabolic perturbation. In all patients, the volume of ultrafiltration was determined by the attending physician.

A Fresenius 4008 H system with biocompatible membranes (Polyflux™; Gambro, Hechingen, Germany) and bicarbonate-buffered dialysate (Fresenius Medical Care SK-F213, Bad Homburg, Germany) at 37°C was used for haemodialysis. Arteriovenous fistulae were used in ambulatory patients, whereas Mahurkar^® ^catheters (Tyco Healthcare Group LP, Mansfield, MA, USA) were used in ICU patients.

Perdialytic hypotension was defined as a drop in systolic blood pressure of greater than 30% or to below 100 mmHg [16]. When hypotension occurred, a bolus of 20 ml hypertonic glucose solution (30%) was injected through the venous line of the haemodialysis circuit, and the ultrafiltration rate was reduced when necessary. Attention was paid to avoid fluid challenges and changes in vasopressor infusion rate throughout the study period.

### Echocardiography Doppler

Transthoracic echocardiography (TTE) was performed in ambulatory patients, whereas transoesophageal echocardiography (TEE) was used in ventilated ICU patients. The echocardiographic study was performed before (baseline) and at least one hour after haemodialysis using a SONOS 5500 upper-end platform (Philips Ultrasound, Andover, MA, USA) equipped with a 2.5 to 4 MHz broadband transducer or a 5 MHz multiplane TEE probe. Respiratory tracing in spontaneously breathing patients or airway pressure curve in ventilated patients were displayed continuously. The same experienced operator performed all TTE and TEE studies and took offline measurements from digitally recorded images. All measurements were performed in triplicate at end-expiration identified on respiratory tracings, and were averaged. Measurements were performed in random order and not consecutively for the same patient, with the investigator being blinded to both body weight and study phase (before or after haemodialysis).

Transmitral Doppler velocities were recorded in the four-chamber view with the sample volume located at the tip of mitral valve leaflets. In all patients, peak E and A wave velocities, and deceleration time of early transmitral flow velocity were measured. Isovolumic relaxation time was only measured in ambulatory patients. Pulsed wave Doppler velocity profile of pulmonary vein flow was obtained in the right upper pulmonary vein using TTE or in the left upper pulmonary vein using TEE. Peak S and D pulmonary vein forward flow velocities were measured. Both mitral early diastole/atrial contraction maximal velocity (E/A) and pulmonary vein systolic/diastolic maximal velocity (S/D) ratios were calculated. Finally, pulsed wave Doppler velocities of aortic flow were recorded at the level of the LV outflow tract in the apical five-chamber view using TTE or in the transgastric longitudinal view (around 120°) with multiplane TEE. Cardiac index was obtained by measuring the velocity-time integral of aortic Doppler tracings and the diameter of the LV outflow tract [20]. In ICU patients, systemic vascular resistance was calculated conventionally.

A 5 mm DTI sample volume was placed at the septal and lateral portions of the mitral annulus to record early diastolic velocity (E' wave) in spectral pulsed mode. To obtain colour M-mode recordings, the width of field was reduced and centred on the mitral valve and LV inflow tract, and colour flow mapping was activated with a Nyquist limit set at around 45 cm/s [21]. M-mode cursor was placed through the centre of the mitral flow and aligned in the direction of the inflow jet. Propagation velocity of LV inflow at early diastole (Vp) was measured as the slope of the first aliasing velocity during early filling, from the mitral valve plane to 4 cm distally into the LV cavity [21]. LV diastolic dysfunction was defined by the presence of a peak E' wave velocity of under 8 cm/s or a Vp under 45 cm/s [22].

LV mass was measured using the method described by Reichek and coworkers [23] and LV hypertrophy was defined as a LV mass index of 143 g/m^2 ^or greater for men and 102 g/m^2 ^for women [24]. LV volumes and ejection fraction were measured in the four-chamber view using the modified Simpson's rule [25]. LV systolic dysfunction was defined as an ejection fraction under 50% at baseline.

### Statistical analysis

Two-dimensional echocardiographic findings and Doppler parameters were compared within the two study groups (haemodialysis and ICU patients) before and after haemodialysis using a Wilcoxon matched pairs test. Haemodialysis patients were divided in two subsets, according to baseline LV systolic function (LV ejection fraction < 50% or ≥ 50%). The same comparison was performed within each subset of haemodialysis patients in order to assess the potential influence of LV systolic performance on ultrafiltration-related variations in Doppler parameters. Values are expressed as mean ± standard deviation. *P *< 0.05 was considered statistically significant. In 20 randomly selected patients, the senior investigator repeated Doppler measurements after a two month interval and another investigator, who was experienced in echocardiography, performed the same measurements to determine their reproducibility. Inter-observed and intra-observer variabilities were calculated as the absolute difference between the two sets of measurements divided by the mean value of measurements, and expressed as a percentage of error. Agreement for the measurement of Doppler parameters was also assessed using the intraclass correlation coefficient.

## Results

No perdialytic hypotension was observed and haemodynamics remained fairly stable throughout the study period in the two patient groups (Table 1). In haemodialysis patients, the greater volume of ultrafiltration tended to further reduce LV end-diastolic volume index compared with ICU patients (16 ± 8 ml/m^2 ^versus 11 ± 4 ml/m^2^; *P *= 0.08). As a result, cardiac index significantly decreased after ultrafiltration in haemodialysis patients but not in ICU patients (Table 1). In ambulatory patients, blood pressure decreased after haemodialysis whereas it remained stable in ICU patients receiving vasopressors, because of a compensatory increase in systemic vascular resistance (Table 1).

**Table 1:** Haemodynamic and echocardiography Doppler findings obtained before and after haemodialysis

	Haemodialysis patients (*n *= 37)	ICU patients (*n *= 8)
Ultrafiltration (l)	3.0 ± 1.1	1.9 ± 0.9^a^
LV mass index (g/m^2^)	102 ± 33	90 ± 13
LV ejection fraction (%)	54 ± 16	49 ± 22
Body weight (kg)^b^	67.5 ± 16.0/64.5 ± 15.3^c^	85.2 ± 19.6/83.4 ± 20.0^c^
Systolic BP (mmHg)^b^	145 ± 17/133 ± 26^c^	135 ± 13/131 ± 22
Diastolic BP (mmHg)^b^	81 ± 10/76 ± 13	73 ± 13/67 ± 9
Heart rate (beats/min)^b^	75 ± 12/77 ± 14	91 ± 23/91 ± 23
Cardiac index (l/min per m^2^)^b^	3.3 ± 0.9/2.9 ± 0.8^c^	3.2 ± 0.6/3.0 ± 0.7
CVP (mmHg)^b^	-/-	13 ± 6/8 ± 6^c^
SVR (dynes·s/cm^5^)^b^	-/-	989 ± 134/1111 ± 235^c^
LVEDVI (ml/m^2^)^b^	73 ± 26/57 ± 26^c^	56 ± 12/45 ± 13^c^
IVRT (ms)^b^	81 ± 16/105 ± 37^c^	-/-
V_max _E (cm/s)^b^	94 ± 25/71 ± 26^c^	77 ± 17/70 ± 22
V_max _A (cm/s)^b^	97 ± 25/94 ± 30	70 ± 32/76 ± 34
E/A ratio^b^	1.00 ± 0.25/0.77 ± 0.26^c^	1.47 ± 1.29/1.06 ± 0.59^c^
DT_E _(ms)^b^	194 ± 58/271 ± 114^c^	147 ± 41/241 ± 92
V_max _S (cm/s)^b^	59 ± 15/59 ± 14	57 ± 22/59 ± 8
V_max _D (cm/s)^b^	51 ± 16/42 ± 11^c^	53 ± 21/55 ± 17
S/D ratio^b^	1.21 ± 0.32/1.44 ± 0.30^c^	1.23 ± 0.58/1.15 ± 0.32

^a^*P *< 0.05 versus haemodialysis patients (Mann-Whitney test). ^b^Values are expressed as before/after haemodialysis. ^c^*P *< 0.05 versus baseline. BP, blood pressure; CVP, central venous pressure; ICU, intensive care unit; IVRT, isovolumic relaxation time; LV, left ventricular; LVEDVI, left ventricular end-diastolic volume index; SVR, systemic vascular resistance; V_max_, maximal velocity; DT_E_, E wave deceleration time.

**Figure 1:**
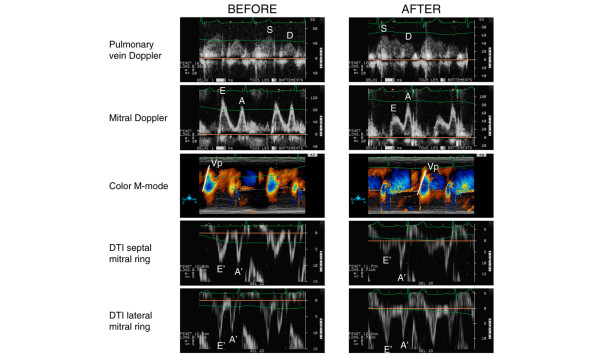
Variations in Doppler velocity profiles resulting from ultrafiltration-related intravascular volume reduction in a haemodialysis patient. Note that ultrafiltration tended to increase the pulmonary vein systolic/diastolic maximal velocity ratio (S/D), decrease the mitral early diastole/atrial contraction maximal velocity ratio (E/A), decrease colour M-mode propagation velocity (Vp;) and decrease Doppler Tissue Imaging E' septal velocity, whereas E' velocity remained fairly constant when recorded on the lateral aspect of the mitral ring.

Despite the absence of relevant tachycardia secondary to ultrafiltration-related intravascular volume reduction, haemodialysis induced substantial alterations in both mitral and pulmonary vein Doppler patterns in the two study groups (Table 1). In haemodialysis patients, isovolumic relaxation time and E-wave deceleration time increased after ultrafiltration. Because the E-wave maximal velocity decreased whereas the A-wave maximal velocity remained unchanged after ultrafiltration, the E/A ratio was significantly reduced by haemodialysis (Figure 1). Opposite variations were observed with the S/D ratio, because the D-wave maximal velocity decreased in parrallel to the E-wave velocity, whereas the S-wave maximal velocity remained unchanged after ultrafiltration. Overall, variations in pulsed wave Doppler indices induced by haemodialysis were less pronounced in ICU patients (Table 1).

DTI and colour M-mode Doppler findings obtained before and after haemodialysis in the two study groups are shown in Table 2. In six patients, Vp was not measured because of inadequate imaging quality, whereas TDI was successfully performed in all studied patients. At baseline, 18 haemodialysis patients (49%) had low E' maximal velocities, consistent with abnormal LV relaxation, whereas Vp was decreased in 14 patients (38%). Among them, eight patients had concentric LV hypertrophy and 10 had LV systolic dysfunction. TDI E' velocity remained stable after ultrafiltration in ICU patients, whereas it decreased significantly only when recorded at the level of the septal mitral annulus in haemodialysis patients (Figure 1). Vp uniformly decreased after ultrafiltration in the two study groups, the difference being significant only in haemodialysis patients (Table 2). Both the E/E' and E/Vp ratios decreased after haemodialysis in ambulatory patients, whereas they were not significantly altered by ultrafiltration in ICU patients (Table 2).

**Table 2:** Tissue Doppler imaging and colour M-mode Doppler findings obtained before and after haemodialysis

	Haemodialysis patients (*n *= 37)		ICU patients (*n *= 8)	
	
	Before haemodialysis	After haemodialysis	Before haemodialysis	After haemodialysis
Heart rate (beats/min)	75 ± 12	77 ± 14	91 ± 23	91 ± 23
V_max _E' lateral (cm/s)	8.9 ± 3.1	8.3 ± 2.6	10.7 ± 2.6	10.5 ± 2.4
V_max _E' septal (cm/s)	7.1 ± 2.5	5.9 ± 1.7^a^	7.9 ± 2.9	7.8 ± 1.6
Vp (cm/s)	45 ± 11	41 ± 13^a^	43 ± 8	36 ± 7
E/E' lateral	12 ± 6	9 ± 5^a^	8 ± 4	7 ± 3
E/E' septal	14 ± 5	13 ± 5^a^	11 ± 5	9 ± 4
E/Vp	2.2 ± 0.6	1.9 ± 0.7^a^	1.9 ± 0.6	2.0 ± 0.6

ICU, intensive care unit; V_max_, maximal velocity; Vp, propagation velocity. ^a^*P *< 0.05 versus baseline.

In haemodialysis patients, baseline LV systolic function apparently failed to alter the effect of ultrafiltration on Doppler parameters of LV diastolic function (Table 3). The volume of ultrafiltration was similar in the two subsets of patients (3.0 ± 1.2 l versus 2.8 ± 0.9 l; *P *= 0.43) and induced a comparable preload reduction, reflected by the close decrease in LV end-diastolic volume index (18 ± 12 ml/m^2 ^versus 20 ± 15 ml/m^2^; *P *= 0.9). DTI E' velocity tended to be less affected by ultrafiltration when measured at the lateral than at the septal aspect of the mitral ring, and variations were less pronounced in the presence of LV systolic dysfunction. In contrast, Vp tended to decrease with preload reduction, regardless of LV systolic function (Table 3).

**Table 3:** Doppler findings obtained before and after ultrafiltration in hemodialysis patients according to their baseline left ventricular systolic function

	Preserved LV systolic function (*n *= 27)	Altered LV systolic function (*n *= 10)
Ultrafiltration (l)	3.0 ± 1.2	2.8 ± 0.9
Mean BP (mmHg)^a^	105 ± 10/97 ± 15^b^	95 ± 10^c^/89 ± 17
LVEDVI (ml/m^2^)^a^	66 ± 12/48 ± 14^b^	92 ± 41^c^/79 ± 41^b^
Cardiac index (L/min per m^2^)^a^	3.6 ± 0.9/3.1 ± 0.7^b^	2.9 ± 0.8^c^/2.6 ± 1.0
HR (beats/min)^a^	76 ± 12/78 ± 14	72 ± 13/74 ± 15
IVRT (ms)^a^	76 ± 13/97 ± 18^b^	95 ± 18^c^/128 ± 61^b^
V_max _E (cm/s)^a^	98 ± 25/73 ± 27^b^	81 ± 22/64 ± 22^b^
V_max _A (cm/s)^a^	99 ± 25/94 ± 31	92 ± 27/96 ± 28
E/A ratio^a^	1.03 ± 0.25/0.80 ± 0.24^b^	0.92 ± 0.25/0.69 ± 0.29^b^
DT_E _(ms)^a^	187 ± 42/273 ± 91^b^	211 ± 90/234 ± 86
V_max _S (cm/s)^a^	64 ± 11/62 ± 12	47 ± 16^c^/50 ± 16
V_max _D (cm/s)^a^	57 ± 15/46 ± 10^b^	36 ± 5^c^/33 ± 9
S/D ratio^a^	1.17 ± 0.28/1.39 ± 0.30	1.31 ± 0.41/1.53 ± 0.32^b^
V_max _E' septal (cm/s)^a^	7.7 ± 2.4/6.2 ± 1.5	5.6^c ^± 2.0/5.0 ± 2.0
V_max _E' lateral (cm/s)^a^	9.5 ± 2.9/8.8 ± 2.5	7.2 ± 2.9/7.0 ± 2.8
Vp (cm/s)^a^	48 ± 8/44 ± 9	35 ± 11^c^/31 ± 17
E/E' lateral^a^	11 ± 5/9 ± 4^b^	13 ± 8/12 ± 7
E/E' septal^a^	14 ± 6/12 ± 5^b^	15 ± 5/14 ± 6
E/Vp^a^	2.1 ± 0.6/1.7 ± 0.6	2.6 ± 0.7^c^/2.3 ± 0.8

^a^Values are expressed as before/after haemodialysis. ^b^*P *< 0.05 versus baseline. ^c^*P *< 0.05 versus patients with preserved LV systolic function, defined as a LV ejection fraction ≥ 50% (Mann-Whitney test). BP, blood pressure; DT_E_, E wave deceleration time; HR, heart rate; IVRT, isovolumic relaxation time; LV, left ventricular; LVEDVI, left ventricular end-diastolic volume index; V_max_, maximal velocity; Vp, propagation velocity.

Inter-observer variability in measurement of Doppler parameters ranged from 1% to 13%, whereas intra-observer variability ranged from 2% to 7%. Notably, measurement of DTI E' velocity at the level of the mitral ring was more reproducible than that of Vp (Table 4).

**Table 4:** Inter-observer and intra-observer variability in Doppler measurements

	IVRT	V_max _E	V_max _A	DT_E_	V_max _S	V_max _D	V_max _E' septal	V_max _E' lateral	Vp
Inter-observer^a^	10%	1%	3%	13%	4%	5%	4%	5%	11%
r^b^	0.34 (-0.13 to +0.68)	0.99 (0.98–1.0)	0.98 (0.95–0.99)	0.31 (-0.15 to +0.66)	0.86 (0.63–0.95)	0.87 (0.65–0.96)	0.97 (0.91–0.99)	0.93 (0.82–0.97)	0.22 (-0.27 to +0.62)
Intra-observer^a^	6%	2%	2%	7%	4%	6%	2%	2%	7%
r^b^	0.85 (0.65–0.94)	0.98 (0.94–0.99)	0.98 (0.95–0.99)	0.72 (0.42–0.88)	0.87 (0.67–0.95)	0.74 (0.41–0.90)	0.93 (0.83–0.97)	0.95 (0.87–0.98)	0.54 (0.12–0.80)

^a^Mean percentage error. ^b^Intraclass coefficient correlation (numbers in parentheses are 95% confidence intervals). IVRT, isovolumic relaxation time; V_max_, maximal velocity; DT_E_, E wave deceleration time; Vp, propagation velocity.

## Discussion

Haemodialysis represents an unparalleled model of abrupt alteration in LV loading conditions. Ultrafiltration allows achievement of marked preload reduction, as reflected in our patients by a significant decrease in LV end-diastolic volume index, a commonly used surrogate of LV preload (Table 1). Importantly, careful reduction of intravascular volume induced by ultrafiltration failed to trigger reflex tachycardia (Table 1), thus avoiding the confounding effect of heart rate on pulsed wave Doppler velocity profiles [1]. In our ICU patients, the absence of blood volume expansion or change in vasopressor infusion rate throughout the study period limited additional variations in LV loading conditions. This allowed us to assess more specifically the effect of haemodialysis-related preload reduction on Doppler indices that are routinely used in the assessment of LV diastolic function [1,22]. Echocardiographic studies were performed immediately before and at least one hour after haemodialysis to reach a relatively stable volaemic state, the process of plasma refilling being completed by that time [16]. Finally, measurements of Doppler parameters were reproducible, as reported earlier in our ICU for two-dimensional measurements [26]. In keeping with the results reported by Bouhemad and coworkers [27], variability in Vp measurement was greater than that in DTI E' velocity in our patients (Table 4).

The high prevalence of LV diastolic dysfunction found at baseline in our patients with end-stage chronic renal failure using previously proposed diagnostic criteria based on DTI and colour M-mode [22] is related to the frequency of LV hypertrophy and congestive heart failure in this population [28,29]. Previous studies reported similar results with use of conventional pulsed wave Doppler [30-32]. Not surprisingly, the present study confirms the load dependence of the pulsed wave Doppler parameters used to evaluated LV diastolic properties. As previously reported [15,16,32-35], the abrupt preload reduction induced by ultrafiltration significantly decreased E-wave maximal velocities and the E/A ratio because A-wave velocities remained unaffected (Table 1). Both the isovolumic relaxation time and E-wave deceleration time were significantly prolonged by volume reduction, as previously shown [14,15,32,33,35]. Finally, the evolution of pulmonary vein Doppler D wave after intravascular volume withdrawal paralleled that of mitral Doppler E wave, and the S/D ratio increased after haemodialysis (Table 1), as was previously reported [14,17]. In light of these findings, the conclusions of clinical studies using conventional pulsed wave Doppler parameters to identify transient LV diastolic dysfunction in acute settings (for instance, septic shock) should be interpreted with caution [5-7].

In the present study, certain recently proposed Doppler parameters of LV diastolic function appeared also to be sensitive to acute and marked variations in LV preload related to ultrafiltration. In haemodialysis patients, TDI E' velocity decreased with preload reduction when recorded at the septal portion of the mitral annulus, as previously reported [16], whereas TDI E' velocities recorded at the lateral aspect of the mitral ring remained unaffected by haemodialysis (Table 2). Similar discrepancies in haemodialysis-related variations in E' maximal velocities according to site of measurement were previously reported [14,17]. As previously observed [16], Vp globally decreased after haemodialysis in ambulatory patients (Table 2).

In our ventilated ICU patients, ultrafiltration-induced variations in pulsed wave Doppler parameters were similar to those observed in haemodialysis patients but were of lesser magnitude (Table 1). Of note, Vp tended to decrease after haemodialysis whereas DTI E' velocity remained fairly stable, regardless of the site of measurement (Table 2). This is presumably related to the lower volume of ultrafiltration withdrawn during haemodialysis from these critically ill patients under vasopressors to limit perdialytic hypotension [36], when compared with our haemodialysis patients. Overall, we found no correlation between the magnitude of variation in DTI velocity or Vp and the volume of ultrafiltration withdrawn from our patients (data not shown). Nevertheless, the amount and rapidity of preload reduction may play a major role in the decreases in E' septal maximal velocity and Vp observed in certain patients, as has previously been demonstrated in other clinical settings [37].

Although of lesser magnitude, a substantial decrease in septal E' velocity and Vp induced by ultrafiltration was observed in the subset of haemodialysis patients with altered LV systolic function (Table 3). In a similar clinical setting, Liu and coworkers [18,19] found that Vp was preload dependent in patients with preserved LV systolic function, whereas it was not significantly altered by haemodialysis in patients with LV systolic dysfunction. This apparently discrepant findings may be attributable to the lower volume of ultrafiltration used in these studies [18,19]. Indeed, small preload variations induced by Valsalva manoeuvre, passive leg lifting and sublingual nitroglycerin fail to alter Vp, regardless of LV systolic function [12]. Animal studies suggested that TDI velocities are partly influenced by preload, but their preload dependence appears to decrease with worsening relaxation and associated LV diastolic dysfunction [38-40]. Although most of our haemodialysis patients had severe LV diastolic dysfunction associated with high filling pressure, as reflected by elevated E/Vp and E/E' ratios [10,21,27,41], most of the Doppler indices of LV diastolic function were significantly altered by the marked preload reduction related to the high volumes of ultrafiltration used in the present study.

The study has several limitations. Our results cannot be extrapolated to clinical settings in which variations in LV loading conditions are less abrupt than those obtained by intermittent haemodialysis. The group of ICU patients was fairly small, and observed results in this specific subset of patients remain to be confirmed. The hypothesis raised by the present data is that smaller changes in Doppler indices observed in ICU patients are presumably related to the lower volume of ultrafiltration withdrawn from these patients compared with haemodialysis patients, rather than a potential lusitropic effect of catecholamines. Finally, we did not assess the potential role of afterload variations and changes in cardiac contractility induced by haemodialysis on observed variations in Doppler indices [42].

## Conclusion

This study confirms the preload dependence of conventional pulsed wave Doppler parameters routinely used to evaluate LV diastolic function. Among the recently proposed Doppler indices for the evaluation of LV diastolic properties, TDI E' maximal velocity measured at the lateral portion of the mitral annulus appeared to be relatively preload independent and reproducible. In contrast, E' septal velocity and Vp appeared sensitive to a marked and abrupt preload reduction. The preload dependence of Doppler parameters was not significantly influenced by LV systolic function. Accordingly, Doppler parameters should be used cautiously to evaluate LV diastolic properties and to diagnose transient diastolic dysfunction in clinical settings characterized by abrupt and marked changes in cardiac loading conditions.

## Key messages

• Abrupt variations in cardiac preload markedly alters conventional pulsed wave Doppler parameters routinely used to assess LV diastolic properties.

• Marked preload reduction may also alter both DTI E' velocity when recorded at the septal mitral ring and Vp measured by colour M-mode.

• DTI E' velocity recorded at the lateral aspect of the mitral ring appears to be relatively insensitive to variations in preload.

• These variations were apparently unaffected by LV systolic function.

## Abbreviations

DTI = Doppler tissue imaging; E/A = mitral early diastole/atrial contraction maximal velocity ratio; E' wave = DTI early diastolic velocity of the mitral annulus; ICU = intensive care unit; LV = left ventricular; S/D = pulmonary vein systolic/diastolic maximal velocity ratio; TEE = transoesophageal echocardiography; TTE = transthoracic echocardiography; Vp = propagation velocity of LV inflow at early diastole measured using color M-mode.

## Competing interests

The authors declare that they have no competing interests.

## Authors' contributions

PV, JCA and HG contributed to the design of the clinical study, data analysis and preparation of the manuscript. VA, JL, JFM and BF contributed to the recruitment and clinical evaluation of patients throughout the study period.
